# Supplementary material for: Cervical spine immobilisation following blunt trauma in pre-hospital and emergency care: A systematic review
Source: PLoS One. 2024 Apr 25;19(4):e0302127. doi: 10.1371/journal.pone.0302127 (PMC11045128; doi:10.1371/journal.pone.0302127)
Supplement: S1 Table — (DOCX) [file pone.0302127.s001.docx]

**S1 Table. Literature search STRATEGIES**

| **Database searched:** | **Ovid MEDLINE(R) Epub Ahead of Print, In-Process & Other Non-Indexed Citations, Ovid MEDLINE(R) Daily, Ovid MEDLINE and Versions(R)** |
| --- | --- |
| **Platform or provider used:** | **Ovid SP** |
| **Date of coverage:** | **1946 to September 2023** |
| **Search undertaken:** | **September 2023** |

1 exp Spinal Injuries/

2 exp Spinal Cord Injuries/

3 exp Neck Injuries/

4 Spine/ or Thorax/ or Neck/ or Cervical Vertebrae/

5 ah.fs.

6 4 and 5)

7 ((spine or spinal or cervical or lumbar or thora* or neck or back) adj3 (injur* or trauma*)).ti,ab.

8 exp Whiplash Injuries/

9 whiplash.ti,ab.

10 exp Spinal Fractures/

11 "c-spine injur*".ti,ab.

12 exp Cervical Vertebrae/in [Injuries]

13 *Spine/

14 (neck adj3 sprain*).ti,ab.

15 or/1-3,6-14

16 exp Immobilization/

17 exp Orthotic Devices/

18 (immobili* or mobili* or stabili*).ti,ab.

19 (collar* or back?board* or spine?board* or spinal?board* or head?block* or sand?bag*).ti,ab.

20 (orthosis or orthotic or protective device* or physical restraint*).ti,ab.

21 (splint* or neutral position or recovery position or strapping or strapped or straps or tapes or taping or log roll*).ti,ab.

22 (kendrick or vacuum mattress* or vacuum stretcher* or scoop stretcher* or support surface*).ti,ab.

23 or/16-22

24 15 and 23

25 exp Emergency Service, Hospital/

26 exp Emergency Medical Services/

27 exp Emergency Medicine/

28 (emergency adj2 service*).ti,ab.

29 "emergency care".ti,ab.

30 "urgent care".ti,ab.

31 "emergency department*".ti,ab.

32 "accident and emergency".ti,ab.

33 casualty.ti,ab.

34 exp Emergency Medical Technicians/

35 exp Emergency Treatment/

36 exp Air Ambulances/

37 exp First Aid/

38 exp Military Medicine/

39 exp Ambulances/

40 (prehospital or pre-hospital or paramedic* or ambulance*).ti,ab.

41 (out-of-hospital or out of hospital).ti,ab.

42 (EMS or EMT).ti,ab.

43 (emergency technician* or emergency practitioner* or emergency dispatch* or emergency despatch* or first responder* or first response).ti,ab.

44 (emergency rescue or emergency resus* or emergency triage).ti,ab.

45 advanced life support.ti,ab.

46 rescuer*.ti,ab.

47 exp Trauma Centers/

48 (trauma adj (patients or victims)).ti,ab.

49 exp "Transportation of Patients"/

50 (transport adj3 patient*).ti,ab.

51 or/25-50

52 24 and 51

53 limit 52 to yr="2015 -Current"

| **Databases searched:** | **EMBASE** |
| --- | --- |
| **Platform or provider used:** | **Ovid SP** |
| **Date of coverage:** | **1974 to September 2023** |
| **Search undertaken:** | **September 2023** |

1     exp spine injury/

2     exp spinal cord injury/

3     exp neck injury/

4     spine/

5     thorax/

6     neck/

7     cervical vertebra/

8     4 or 5 or 6 or 7

9     (injur* or trauma*).ti,ab.

10     8 and 9 (16918)

11     exp whiplash injury/

12     whiplash.ti,ab.

13     exp spine fracture/

14     "c-spine injur*".ti,ab.

15     *spine/

16     (neck adj3 sprain*).ti,ab.

17     or/1-3,10-16

18     exp immobilization/

19     exp orthosis/

20     exp cervical spine immobilization device/

21     (immobili* or mobili* or stabili*).ti,ab.

22     (collar* or back?board* or spine?board* or spinal?board* or head?block* or sand?bag*).ti,ab.

23     (orthosis or orthotic or protective device* or physical restraint*).ti,ab.

24     (splint* or neutral position or recovery position or strapping or strapped or straps or tapes or taping or log roll*).ti,ab.

25     (kendrick or vacuum mattress* or vacuum stretcher* or scoop stretcher* or support surface*).ti,ab.

26     or/18-25

27     17 and 26

28     exp hospital emergency service/

29     exp emergency health service/

30     exp emergency medicine/

31     (emergency adj2 service*).ti,ab.

32     "emergency care".ti,ab.

33     "urgent care".ti,ab.

34     "emergency department*".ti,ab.

35     "accident and emergency".ti,ab.

36     casualty.ti,ab.

37     exp rescue personnel/

38     exp emergency treatment/

39     exp air medical transport/

40     exp first aid/

41     exp military medicine/

42     exp ambulance/

43     (prehospital or pre-hospital or paramedic* or ambulance*).ti,ab.

44     (out-of-hospital or out of hospital).ti,ab.

45     (EMS or EMT).ti,ab.

46     (emergency technician* or emergency practitioner* or emergency dispatch* or emergency despatch* or first responder* or first response).ti,ab.

47     (emergency rescue or emergency resus* or emergency triage).ti,ab.

48     advanced life support.ti,ab.

49     rescuer*.ti,ab.

50     (trauma adj (patients or victims)).ti,ab.

51     exp patient transport/

52     (transport adj3 patient*).ti,ab.

53     or/28-52

54     27 and 53

55     limit 54 to yr="2015 -Current"

| **Databases searched:** | **CINAHL** |
| --- | --- |
| **Platform or provider used:** | **EBSCO** |
| **Date of coverage:** | **1981 to September 2023** |
| **Search undertaken:** | **September 2023** |

S1 (MH "Spinal Injuries+")

S2 (MH "Spinal Cord Injuries+")

S3 (MH "Neck Injuries+")

S4 (MH "Spine/AH")

S5 (MH "Thorax/AH")

S6 (MH "Neck/AH")

S7 (MH "Cervical Vertebrae/AH")

S8 ((spine or spinal or cervical or lumbar or thora* or neck or back) N3 (injur* or trauma*))

S9 (MH "Whiplash Injuries")

S10 whiplash

S11 (MH "Spinal Fractures+")

S12 "c-spine injur*"

S13 (MH "Cervical Vertebrae+/IN")

S14 (MM "Spine")

S15 (neck N3 sprain*)

S16 S1 OR S2 OR S3 OR S4 OR S5 OR S6 OR S7 OR S8 OR S9 OR S10 OR S11 OR S12 OR S13 OR S14 OR S15

S17 (MH "Immobilization")

S18 (MH "Orthoses+")

S19 (immobili* or mobili* or stabili*)

S20 (collar* or back?board* or spine?board* or spinal?board* or head?block* or sand?bag*)

S21 (orthosis or orthotic or protective device* or physical restraint*)

S22 (splint* or neutral position or recovery position or strapping or strapped or straps or tapes or taping or log roll*)

S23 (kendrick or vacuum mattress* or vacuum stretcher* or scoop stretcher* or support surface*)

S24 S17 OR S18 OR S19 OR S20 OR S21 OR S22 OR S23

S25 S16 AND S24

S26 "Emergency Service, Hospital"

S27 (MH "Emergency Medical Services+")

S28 (MH "Emergency Medicine")

S29 (emergency N2 service*)

S30 "emergency care"

S31 "urgent care"

S32 "emergency department*"

S33 "accident and emergency"

S34 casualty

S35 (MH "Emergency Medical Technicians")

S36 (MH "Emergency Treatment+")

S37 "Air Ambulances"

S38 (MH "First Aid")

S39 (MH "Military Medicine")

S40 (MH "Ambulances")

S41 (prehospital or pre-hospital or paramedic* or ambulance*)

S42 (out-of-hospital or out of hospital)

S43 (EMS or EMT)

S44 (emergency technician* or emergency practitioner* or emergency dispatch* or emergency despatch* or first responder* or first response)

S45 (emergency rescue or emergency resus* or emergency triage)

S46 advanced life support

S47 rescuer*

S48 (MH "Trauma Centers")

S49 (trauma N1 (patients or victims))

S50 (MH "Transportation of Patients+")

S51 (transport N3 patient*)

S52 S26 OR S27 OR S28 OR S29 OR S30 OR S31 OR S32 OR S33 OR S34 OR S35 OR S36 OR S37 OR S38 OR S39 OR S40 OR S41 OR S42 OR S43 OR S44 OR S45 OR S46 OR S47 OR S48 OR S49 OR S50 OR S51

S53 S25 AND S52

S54 S25 AND S52 Limiters - Published Date: 20150101-20230919

| **Databases searched:** | **Cochrane CENTRAL Register of Randomised Controlled Trials & Cochrane Database of Systematic Reviews** |
| --- | --- |
| **Platform or provider used:** | **www.thecochranelibrary.com** |
| **Date of coverage:** | **Inception to September 2023** |
| **Search undertaken:** | **September 2023** |

#1 MeSH descriptor: [Spinal Injuries] explode all trees

#2 MeSH descriptor: [Spinal Cord Injuries] explode all trees

#3 MeSH descriptor: [Neck Injuries] explode all trees

#4 MeSH descriptor: [Spine] this term only

#5 MeSH descriptor: [Thorax] this term only

#6 MeSH descriptor: [Neck] this term only

#7 MeSH descriptor: [Cervical Vertebrae] this term only

#8 #4 or #5 or #6 or #7

#9 injur* or trauma*

#10 #8 and #9

#11 ((spine or spinal or cervical or lumbar or thora* or neck or back) NEAR/3 (injur* or trauma*))

#12 MeSH descriptor: [Whiplash Injuries] explode all trees

#13 whiplash

#14 MeSH descriptor: [Spinal Fractures] explode all trees

#15 "c-spine injur*"

#16 MeSH descriptor: [Spine] this term only

#17 (neck NEAR/3 sprain*)

#18 (OR #1-#3, #10-#17)

#19 MeSH descriptor: [Immobilization] explode all trees

#20 MeSH descriptor: [Orthotic Devices] explode all trees

#21 (immobili* or mobili* or stabili*)

#22 (collar* or back?board* or spine?board* or spinal?board* or head?block* or sand?bag*)

#23 (orthosis or orthotic or protective device* or physical restraint*)

#24 (splint* or neutral position or recovery position or strapping or strapped or straps or tapes or taping or log roll*)

#25 (kendrick or vacuum mattress* or vacuum stretcher* or scoop stretcher* or support surface*)

#26 (OR #19-#25)

#27 #18 AND #26

#28 MeSH descriptor: [Emergency Service, Hospital] explode all trees

#29 MeSH descriptor: [Emergency Medical Services] explode all trees

#30 MeSH descriptor: [Emergency Medicine] explode all trees

#31 (emergency NEAR/2 service*)

#32 "emergency care"

#33 "urgent care"

#34 "emergency department*"

#35 "accident and emergency"

#36 casualty

#37 MeSH descriptor: [Emergency Medical Technicians] explode all trees

#38 MeSH descriptor: [Emergency Treatment] explode all trees

#39 MeSH descriptor: [Air Ambulances] explode all trees

#40 MeSH descriptor: [First Aid] explode all trees

#41 MeSH descriptor: [Military Medicine] explode all trees

#42 MeSH descriptor: [Ambulances] explode all trees

#43 (prehospital or pre-hospital or paramedic* or ambulance*)

#44 (out-of-hospital or out of hospital)

#45 (EMS or EMT)

#46 (emergency technician* or emergency practitioner* or emergency dispatch* or emergency despatch* or first responder* or first response)

#47 (emergency rescue or emergency resus* or emergency triage)

#48 advanced life support

#49 rescuer*

#50 MeSH descriptor: [Trauma Centers] explode all trees

#51 (trauma adj (patients or victims))

#52 MeSH descriptor: [] explode all trees

#53 (transport adj3 patient*)

#54 (OR #28-#53)

#55 #27 AND #54 with Cochrane Library publication date Between Jan 2015 and Sept 2023
