# Supplementary material for: Cervical spine immobilisation following blunt trauma in pre-hospital and emergency care: A systematic review
Source: PLoS One. 2024 Apr 25;19(4):e0302127. doi: 10.1371/journal.pone.0302127 (PMC11045128; doi:10.1371/journal.pone.0302127)
Supplement: S2 Table — (DOCX) [file pone.0302127.s002.docx]

**S2 Table List of excluded studies with rationale**

|  | **Authors, year** | **Reason for exclusion** |
| --- | --- | --- |
|  | Ahmed et al., 2018 [1] | No relevant/useable outcome data |
|  | Ala et al., 2016 [2] | Inappropriate intervention/ comparator (before/after removal of cervical collar) |
|  | Baker et al., 2023 [3] | Inappropriate intervention/ comparator (compares rigid versus soft collar immobilisation) |
|  | Backer et al., 2022 [4] | Inappropriate study design/ publication type (review) |
|  | Beeharry et al., 2021 [5] | Inappropriate study design/ publication type (review) |
|  | Bravi et al., 2020 [6] | Inappropriate intervention/ comparator (before/after removal of cervical collar) |
|  | Browne et al., 2021 [7] | No relevant/useable outcome data |
|  | Burch and Olympia, 2017 [8] | No relevant/useable outcome data (abstract only) |
|  | Carmichael et al., 2021 [9] | Inappropriate intervention/ comparator (assesses suitability of Canadian C-Spine Rule for C-spine immobilisation) |
|  | Carter et al., 2016 [10] | Inappropriate study design/ publication type (review) |
|  | Castro-Marin et al., 2020 [11] | Inappropriate intervention/ comparator (no details provided) |
|  | Chang et al., 2017 [12] | Inappropriate intervention/ comparator (assesses EMS provider beliefs regarding spinal immobilisation) |
|  | Chen et al., 2022 [13] | Not cervical spine immobilisation following blunt trauma in pre-hospital and emergency care |
|  | Clemency et al., 2021 [14] | Not cervical spine immobilisation following blunt trauma in pre-hospital and emergency care |
|  | Clemency et al., 2018 [15] | Not cervical spine immobilisation following blunt trauma in pre-hospital and emergency care |
|  | Coggins et al., 2019 [16] | No relevant/useable outcome data |
|  | Colak and Celik, 2020 [17] | Not cervical spine immobilisation following blunt trauma in pre-hospital and emergency care |
|  | Dodd et al., 1995 [18] | Not cervical spine immobilisation following blunt trauma in pre-hospital and emergency care |
|  | Domeier et al., 2005 [19] | Not cervical spine immobilisation following blunt trauma in pre-hospital and emergency care |
|  | Eisner et al., 2021 [20] | Inappropriate study design/ publication type (review) |
|  | Gaither et al., 2018 [21] | No relevant/useable outcome data (abstract only) |
|  | Geldenhuys and Downing, 2020 [22] | Inappropriate study design/ publication type (review) |
|  | Grabel et al., 2018 [23] | Not cervical spine immobilisation following blunt trauma in pre-hospital and emergency care |
|  | Graham et al., 2020 [24] | Not cervical spine immobilisation following blunt trauma in pre-hospital and emergency care |
|  | Gutierrez et al., 2019 [25] | Not cervical spine immobilisation following blunt trauma in pre-hospital and emergency care |
|  | Ham et al., 2014 [26] | Non comparative study |
|  | Ham et al., 2017 [27] | Non comparative study (assesses risk factors for developing pressure ulcers from spinal immobilization with backboard, extrication collar and head blocks) |
|  | Ham et al., 2016 [28] | Non comparative study (AE from spinal immobilization with a CC and head blocks) |
|  | Ham et al., 2017 [29] | Non comparative study (AE from spinal immobilization with a CC and head blocks) |
|  | Hasandarras et al., 2023 [30] | Not cervical spine immobilisation following blunt trauma in pre-hospital and emergency care |
|  | Hodgett and Ward, 2020 [31] | Inappropriate study design/ publication type (review) |
|  | Hood and Considine, 2015 [32] | Inappropriate study design/ publication type (review) |
|  | Huang et al., 2018 [33] | Non comparative study |
|  | Hunt et al., 2001 [34] | Not cervical spine immobilisation following blunt trauma in pre-hospital and emergency care |
|  | Jadgal et al., 2021 [35] | Not cervical spine immobilisation following blunt trauma in pre-hospital and emergency care |
|  | Jones Rhodes et al., 2016 [36] | Non comparative study (survey of EMS professionals) |
|  | Khetarpal et al., 2021 [37] | Non comparative study (survey of EMS professionals) |
|  | Kim et al., 2006 [38] | No relevant/useable outcome data (also foreign language) |
|  | Kolb et al., 1999 [39] | Not cervical spine immobilisation following blunt trauma in pre-hospital and emergency care |
|  | Kreinest et al., 2016 [40] | Non comparative study (development of protocol to support decision-making for spinal immobilisation) |
|  | Kreinest et al., 2017 [41] | Non comparative study (survey of paramedics pre-hospital assessment and treatment of patients suffering spine injury) |
|  | Kulas et al., 2020 [42] | No relevant/useable outcome data (abstract only) |
|  | Linsenmaier et al., 2016 [43] | Not cervical spine immobilisation following blunt trauma in pre-hospital and emergency care (post CT-imaging) |
|  | Mahshidfar et al., 2013 [44] | Inappropriate intervention/ comparator (Not specific to C spine immobilsation) |
|  | McDonald et al., 2021 [45] | Not cervical spine immobilisation following blunt trauma in pre-hospital and emergency care |
|  | Nakanishi et al., 2019 [46] | Inappropriate intervention/ comparator (compares cervical collar use >24 hours vs. <=24hours) |
|  | Nolte et al., 2022 [47] | Non comparative study (development of protocol to support decision-making for spinal immobilisation; same as Kreinest et al.[40]) |
|  | Nolte et al., 2020 [48] | Non comparative study (development of protocol to support decision-making for spinal immobilisation; same as Kreinest et al.[40]) |
|  | Oosterwold et al., 2017 [49] | Non comparative study (assesses characteristics and AE of full/partial spinal immobilisation) |
|  | Oteir et al., 2015 [50] | Inappropriate study design/ publication type (review) |
|  | Ottosen et al., 2019 [51] | Non comparative study (survey of patient experience of spinal immobilisation [cervical collar and spine board] following trauma) |
|  | Purvis et al., 2017 [52] | Inappropriate study design/ publication type (review) |
|  | Serigano et al., 2021 [53] | Inappropriate study design/ publication type (commentary) |
|  | Shrier et al., 2015 [54] | Non comparative study (assess paramedics ability to limit cervical spine motion with 2 stabilisation techniques [head vs. trap squeeze] and transfer methods) |
|  | Stroh and Braude, 2001 [55] | Non comparative study (assesses FKM EMS protocols for selective spine immobilisation for identifying cervical injury) |
|  | Tatum et al., 2017 [56] | Not cervical spine immobilisation following blunt trauma in pre-hospital and emergency care |
|  | Thompson et al., 2021 [57] | Non comparative study (survey exploring the views and perspectives of pre-hospital care providers about immobilising patients) |
|  | Trentzsch et al., 2023 [58] | Inappropriate study design/ publication type (review) |
|  | Tsutsumi et al., 2018 [59] | No relevant useable outcome data (and patients with on-scene cardiac arrest owing to blunt trauma only) |
|  | Weber et al. 2015 [60] | Healthy volunteers |

**REFERENCES (S2 Table - List of excluded studies with rationale)**

1. Ahmed OZ, Webman RB, Sheth PD, Donnenfield JI, Yang J, Sarcevic A, et al. Errors in cervical spine immobilization during pediatric trauma evaluation. Journal of Surgical Research. 2018;228:135-41.

2. Ala A, Shams-Vahdati S, Taghizadieh A, Miri SH, Kazemi N, Hodjati SR, et al. Cervical collar effect on pulmonary volumes in patients with trauma. European Journal of Trauma & Emergency Surgery. 2016;42(5):657-60.

3. Baker R, Klim S, Poonian J, Ritchie P, Ng S, Kelly AM. SOFTLY: Comparison of outcomes of rigid versus soft collar during emergency department investigation for potential cervical spine injury in low-risk blunt trauma patients - A pilot study. Emergency Medicine Australasia. 2023;35(4):652-6.

4. Backer HC, Elias P, Braun KF, Johnson MA, Turner P, Cunningham J. Cervical immobilization in trauma patients: soft collars better than rigid collars? A systematic review and meta-analysis. European Spine Journal. 2022;31(12):3378-91.

5. Beeharry MW, Moqeem K, Rohilla MU. Management of Cervical Spine Fractures: A Literature Review. Cureus. 2021;13(4):e14418.

6. Bravi N, Marchetti L, Musoiesi S. Studio osservazionale in merito alla validità della Canadian C-Spine Rule in ambito extra ospedaliero. SCENARIO: Official Italian Journal of ANIARTI. 2020;37(4):43-9.

7. Browne LR, Ahmad FA, Schwartz H, Wallendorf M, Kuppermann N, Lerner EB, et al. Prehospital Factors Associated With Cervical Spine Injury in Pediatric Blunt Trauma Patients. Academic Emergency Medicine. 2021;28(5):553-61.

8. Burch K, Olympia R. Compliance of emergency medical services providers with new prehospital spinal immobilization protocols. Academic Emergency Medicine. 2017;24(Supplement 1):S198.

9. Carmichael H, Vaillancourt C, Shrier I, Charette M, Hobden E, Stiell IG. Evaluating the paramedic application of the prehospital Canadian C-Spine Rule in sport-related injuries. CJEM Canadian Journal of Emergency Medical Care. 2021;23(3):356-64.

10. Carter A, Jensen J, Greene J, Goldstein J, Cook J, Swain J, et al. State of the evidence for Emergency Medical Services (EMS) care of blunt spinal trauma: an analysis of appraised research from the Canadian Prehospital Evidence-based Practice (PEP) Project. CMAJ Canadian Medical Association Journal. 2016;59(3 Supplement 1):S25.

11. Castro-Marin F, Gaither JB, Rice AD, N. Blust R, Chikani V, Vossbrink A, et al. Prehospital Protocols Reducing Long Spinal Board Use Are Not Associated with a Change in Incidence of Spinal Cord Injury. Prehospital Emergency Care. 2020;24(3):401-10.

12. Chang CD, Crowe RP, Bentley MA, Janezic AR, Leonard JC. EMS Providers' Beliefs Regarding Spinal Precautions for Pediatric Trauma Transport. Prehospital Emergency Care. 2017;21(3):344-53.

13. Chen HA, Hsu ST, Shin SD, Jamaluddin SF, Son DN, Hong KJ, et al. A multicenter cohort study on the association between prehospital immobilization and functional outcome of patients following spinal injury in Asia. Scientific Reports. 2022;12(1):3492.

14. Clemency BM, Natalzia P, Innes J, Guarino S, Welch JV, Haghdel A, et al. A Change from a Spinal Immobilization to a Spinal Motion Restriction Protocol was Not Associated with an Increase in Disabling Spinal Cord Injuries. Prehospital & Disaster Medicine. 2021;36(6):708-12.

15. Clemency BM, Tanski CT, Gibson Chambers J, O'Brien M, Knapp AS, Clark AJ, et al. Compulsory Use of the Backboard is Associated with Increased Frequency of Thoracolumbar Imaging. Prehospital Emergency Care. 2018;22(4):506-10.

16. Coggins A, Ebrahimi N, Kemp U, O'Shea K, Fusi M, Murphy M. A prospective evaluation of cervical spine immobilisation in low-risk trauma patients at a tertiary Emergency Department. Australasian Emergency Care. 2019;22(2):69-75.

17. Colak T, Celik K. The Association between Cervical Collar and Intracranial Pressure Measured by the Optic Nerve Sheath Diameter in Trauma Patients Refered to the Emergency Department. Signa Vitae. 2020;16(1):89-95.

18. Dodd F, Simon E, McKeown D, Patrick M. The effect of a cervical collar on the tidal volume of anaesthetised adult patients. Anaesthesia. 1995;50(11):961-3.

19. Domeier RM, Frederiksen SM, Welch K. Prospective performance assessment of an out-of-hospital protocol for selective spine immobilization using clinical spine clearance criteria. Annals of emergency medicine. 2005;46(2):123-31.

20. Eisner ZJ, Delaney PG, Widder P, Aleem IS, Tate DG, Raghavendran K, et al. Prehospital care for traumatic spinal cord injury by first responders in 8 sub-Saharan African countries and 6 other low- and middle-income countries: A scoping review. African Journal Of Emergency Medicine. 2021;11(3):339-46.

21. Gaither JB, Fletcher P, Rice A, Castro-Marin F, Blust R, Chikani V, et al. Does an emergency medical services protocol decrease spinal board use in patients with spinal cord injury? Academic Emergency Medicine. 2018;25(Supplement 1):S21.

22. Geldenhuys MJ, Downing C. Evidence-Based Nursing Care for Spinal Nursing Immobilization: A Systematic Review. Journal of Emergency Nursing. 2020;46(3):318-37.

23. Grabel ZJ, Armaghani SJ, Vu C, Jain A, Yoon ST. Variations in Treatment of C2 Fractures by Time, Age, and Geographic Region in the United States: An Analysis of 4818 Patients. World Neurosurgery. 2018;113:e535-e41.

24. Graham B, Johnson GM, Gurney JM, Shackelford SA, Howard JT, Janak JC. How the Implementation of a Battlefield Clinical Practice Guideline Affects Documentation Practice and Informs Future Research Initiatives: Spinal Injuries and Cervical Collars. Military medicine. 2020;185(7-8):e1209-e15.

25. Gutierrez X, April M, Maddry J, Hill G, Becker T, Schauer S. Incidence of Pediatric Cervical Spine Injuries in Iraq and Afghanistan. Southern Medical Journal. 2019;112(5):271-5.

26. Ham HW, Schoonhoven LL, Galer AA, Shortridge-Baggett LL. Cervical collar-related pressure ulcers in trauma patients in intensive care unit. J Trauma Nurs. 2014;21(3).

27. Ham HW, Schoonhoven LL, Schuurmans MM, Leenen LL. Pressure ulcer development in trauma patients with suspected spinal injury; the influence of risk factors present in the Emergency Department. International emergency nursing. 2017;30:13-9.

28. Ham WH, Schoonhoven L, Schuurmans MJ, Leenen LP. Pressure ulcers, indentation marks and pain from cervical spine immobilization with extrication collars and headblocks: An observational study. Injury. 2016;47(9):1924-31.

29. Ham WH, Schoonhoven L, Schuurmans MJ, Leenen LP. Pressure ulcers in trauma patients with suspected spine injury: a prospective cohort study with emphasis on device-related pressure ulcers. International Wound Journal. 2017;14(1):104-11.

30. Hasandarras AKH, Strandvik GF, Faramawy AE, Areibi NN, Younis B, Mekkodothil A, et al. Intensive Care Physician-Led Clearance of the Cervical Spine: A Retrospective Review of the Utility of a Normal Cervical CT Scan for Safe Removal of Hard Collars by Critical Care Physicians. J Intensive Care Med. 2023:8850666231194529.

31. Hodgett R, Ward R. Are cervical collars effective and safe in prehospital spinal cord injury management? Journal of Paramedic Practice. 2020;12(2):67-78. doi: 10.12968/jpar.2020.12.2.67.

32. Hood N, Considine J. Spinal immobilisaton in pre-hospital and emergency care: A systematic review of the literature. Australasian Emergency Nursing Journal. 2015;18(3):118-37.

33. Huang H, Blecher G, Egerton-Warburton D, Meek R. Current imaging and management practices for suspected cervical spine injury in Emergency Departments: A prospective cohort study. EMA - Emergency Medicine Australasia. 2018;30(Supplement 1):48-9.

34. Hunt K, Hallworth S, Smith M. The effects of rigid collar placement on intracranial and cerebral perfusion pressures. Anaesthesia. 2001;56(6):511-3.

35. Jadgal N, Nikravan Mofrad M, Jamsahar M, Nasiri M. The Clinical Skills of Emergency Medical Service (EMS) Personnel Regarding Spinal Immobilization of Trauma Victims; a Cross Sectional Study. Archives of Academic Emergency Medicine. 2021;9(1):e3.

36. Jones Rhodes W, Steinbruner D, Finck L, Flarity K. Community Implementation of a Prehospital Spinal Immobilization Guideline. Prehospital Emergency Care. 2016;20(6):792-7.

37. Khetarpal S, Smith J, Weiss B, Bhattarai B, Sinha M. Pediatric Cervical Spine Clearance and Immobilization Practice Among Prehospital Emergency Medical Providers: A Statewide Survey. Pediatric Emergency Care. 2021;37(8):e474-e8.

38. Kim YJ, Park JO, Ahn KO, Song KJ, Shin SD, Suh GJ. Validity of Clinical Spine Clearance Criteria for Selective Pre-hospital Spine Immobilization. Journal of the korean society of emergency medicine. 2006;17(4):300‐7.

39. Kolb JC, Summers RL, Galli RL. Cervical collar-induced changes in intracranial pressure. Am J Emerg Med. 1999;17(2):135-7.

40. Kreinest M, Gliwitzky B, Schuler S, Grutzner PA, Munzberg M. Development of a new Emergency Medicine Spinal Immobilization Protocol for trauma patients and a test of applicability by German emergency care providers. Scandinavian Journal of Trauma, Resuscitation & Emergency Medicine. 2016;24:71.

41. Kreinest M, Goller S, Gliwitzky B, Grutzner PA, Kuffer M, Haske D, et al. Expertise of German paramedics concerning the prehospital treatment of patients with spinal trauma. European Journal of Trauma & Emergency Surgery. 2017;43(3):371-6.

42. Kulas M, Brueton-Campbell L, Weldon E, McDonald N, Pryce R. Quantification of head-neck motion in trauma patients in the emergency department under spinal motion restriction: a prospective observational study. Canadian Journal of Emergency Medicine. 2020;22(Supplement 1):S47.

43. Linsenmaier U, Deak Z, Krtakovska A, Ruschi F, Kammer N, Wirth S, et al. Emergency radiology: straightening of the cervical spine in MDCT after trauma--a sign of injury or normal variant? British Journal of Radiology. 2016;89(1061):20150996.

44. Mahshidfar B, Mofidi M, Yari AR, S. M. Long backboard versus vacuum mattress splint to immobilize whole spine in trauma victims in the field: a randomized clinical trial. Prehosp Disaster Med. 2013;28(5):462-5.

45. McDonald N, Kriellaars D, Weldon E, Pryce R. Head-Neck Motion in Prehospital Trauma Patients under Spinal Motion Restriction: A Pilot Study. Prehospital Emergency Care. 2021;25(1):117-24.

46. Nakanishi T, Mitra B, Ackland H, O'Reilly G, Cameron P. Time in Collars and Collar-Related Complications in Older Patients. World Neurosurgery. 2019;129:e478-e84.

47. Nolte PC, Liao S, Kuch M, Grutzner PA, Munzberg M, Kreinest M. Development of a New Emergency Medicine Spinal Immobilization Protocol for Pediatric Trauma Patients and First Applicability Test on Emergency Medicine Personnel. Pediatric Emergency Care. 2022;38(1):e75-e84.

48. Nolte PC, Uzun DD, Liao S, Kuch M, Grutzner PA, Munzberg M, et al. Development and first application testing of a new protocol for preclinical spinal immobilization in children : Assessment of indications based on the E.M.S. IMMO Protocol Pediatric. Unfallchirurg. 2020;123(4):289-301.

49. Oosterwold JT, Sagel DC, van Grunsven PM, Holla M, de Man-van Ginkel J, Berben S. The characteristics and pre-hospital management of blunt trauma patients with suspected spinal column injuries: a retrospective observational study. European Journal of Trauma & Emergency Surgery. 2017;43(4):513-24.

50. Oteir AO, Smith K, Stoelwinder JU, Middleton J, Jennings PA. Should suspected cervical spinal cord injury be immobilised?: a systematic review. Injury. 2015;46(4):528-35.

51. Ottosen CI, Steinmetz J, Larsen MH, Baekgaard JS, Rasmussen LS. Patient experience of spinal immobilisation after trauma. Scandinavian Journal of Trauma, Resuscitation & Emergency Medicine. 2019;27(1):70.

52. Purvis TA, Carlin B, Driscoll P. The definite risks and questionable benefits of liberal pre-hospital spinal immobilisation. American Journal of Emergency Medicine. 2017;35(6):860-6.

53. Serigano O, Riscinti M, Zehtabchi S. Cervical Spine Motion Restriction After Blunt Trauma. Academic Emergency Medicine. 2021;28(4):472-4. doi: 10.1111/acem.14134.

54. Shrier I, Boissy P, Lebel K, Boulay J, Segal E, Delaney JS, et al. Cervical Spine Motion during Transfer and Stabilization Techniques. Prehospital emergency care. 2015;19(1):116‐25.

55. Stroh G, Braude D. Can an out-of-hospital cervical spine clearance protocol identify all patients with injuries? An argument for selective immobilization. Ann Emerg Med. 2001;37(6):609-15.

56. Tatum JM, Dhillon NK, Ko A, Smith EJT, Melo N, Barmparas G, et al. Refusal of cervical spine immobilization after blunt trauma: Implications for initial evaluation and management: A retrospective cohort study. International Journal Of Surgery. 2017;48:228-31.

57. Thompson L, Shaw G, Bates C, Hawkins C, McClelland G, McMeekin P. To collar or not to collar. Views of pre-hospital emergency care providers on immobilisation without cervical collars: a focus group study. British Paramedic Journal. 2021;6(1):38-45.

58. Trentzsch H. Immobilization of the cervical spine in the prehospital phase. [German]. Notfall und Rettungsmedizin. 2023;26(4):275-81.

59. Tsutsumi Y, Fukuma S, Tsuchiya A, Ikenoue T, Yamamoto Y, Shimizu S, et al. Association between spinal immobilization and survival at discharge for on-scene blunt traumatic cardiac arrest: A nationwide retrospective cohort study. Injury. 2018;49(1):124-9.

60. Weber SR, Rauscher P, Winsett RP. Comparison of a Padded Patient Litter and Long Spine Board for Spinal Immobilization in Air Medical Transport. Air Medical Journal. 2015;34(4):213-7.
