# Supplementary material for: Cervical spine immobilisation following blunt trauma in pre-hospital and emergency care: A systematic review
Source: PLoS One. 2024 Apr 25;19(4):e0302127. doi: 10.1371/journal.pone.0302127 (PMC11045128; doi:10.1371/journal.pone.0302127)
Supplement: S3 Table — (DOCX) [file pone.0302127.s003.docx]

**S3 Table. Summary of each study’s risk of bias using the ROBINS-I tool – Review authors’ judgements in detail**

| **Author, year** | **Risk of Bias Domains** | | | | | | | |
| --- | --- | --- | --- | --- | --- | --- | --- | --- |
|  | **Confounding** | **Participant selection** | **Classification of interventions** | **Deviations of intended interventions** | **Missing data** | **Measurement of outcomes** | **Selection of reported results** | **Overall** |
| Asha et al. 2021 [25] | 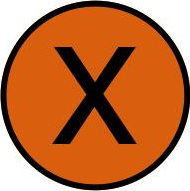 | 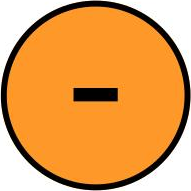 | 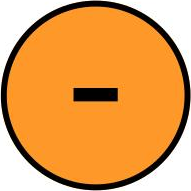 | 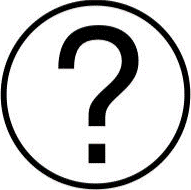 | 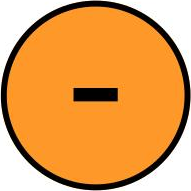 | 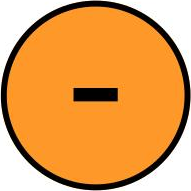 | 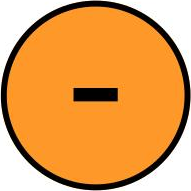 | Serious |
|  | Assume at least, one known important domain was not controlled (adjusted) e.g., MoI, injury severity etc | States 'All patients evaluated for potential traumatic cervical spine injury in the ED were eligible. However, authors state potential for missing patients. | Intervention defined; and determined retrospectively | No information is reported on whether there is deviation from the intended intervention. However, authors state “patients in whom there was concern for cervical spine injury but had their spine cleared by clinical assessment without ever having a collar fitted, or staff failed to add them to the soft collar register” | Missing data for some outcomes reported; measures undertaken to minimise errors and dealing with missing data and all eligible participants accounted for | Assume methods of outcome assessment will be comparable across intervention groups; and  and outcome measure minimally influenced by knowledge of the intervention received by study participants (no indication of blinding); | Outcome measures generally well defined and no indication of selection of results from multiple analyses | At least one domain at serious risk |
| Hauswald et al. 1998 [23] | 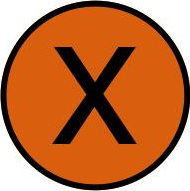 | 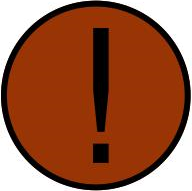 | 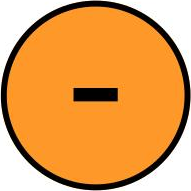 | 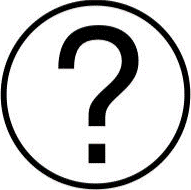 | 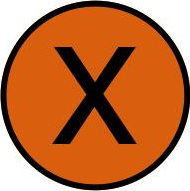 | 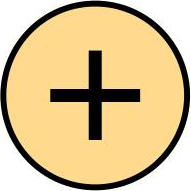 | 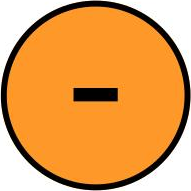 | Critical |
|  | At least, one known important domain was not appropriately measured, or not controlled (adjusted) e.g. injury severity | Different spinal injury populations from two independent locations/ different management and not adjusted in analyses. Whole of eligible “immobilisation for possible C spine injury“ populations not studied | Intervention defined; and determined retrospectively | No information is reported on whether there is deviation from the intended intervention. | Participants with missing data excluded; no details across interventions and or undertaken appropriate analysis | Assume methods of outcome assessment will be comparable across intervention groups; and  and outcome assessors were unaware of the intervention received by study participants; | Outcome measures generally well defined and no indication of selection of results from multiple analyses | At least one domain at critical risk |
| Leonard et al. 2012 [26] | 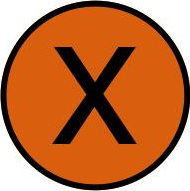 | 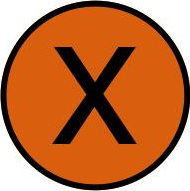 | 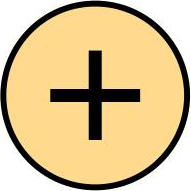 | 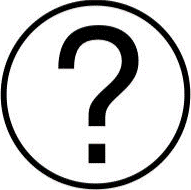 | 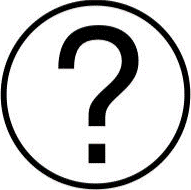 | 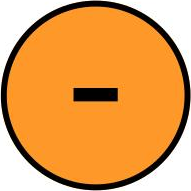 | 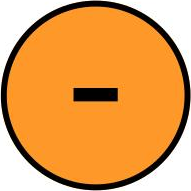 | Serious |
|  | Assume at least, one known important domain was not controlled (adjusted) e.g. MoI, injury severity etc | Selected population; children <18 years; single centre, USA; convenience sample so potential for sampling bias | Intervention defined; and determined prospectively | No information is reported on whether there is deviation from the intended intervention. | No information is reported about missing data or the potential for data to be missing | Assume methods of outcome assessment will be comparable across intervention groups; and  and outcome measure minimally influenced by knowledge of the intervention received by study participants (no indication of blinding); | Outcome measures generally well defined and no indication of selection of results from multiple analyses | At least one domain at serious risk |
| Lin et al. 2011 [27] | 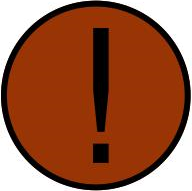 | 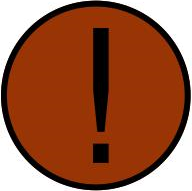 | 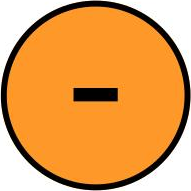 | 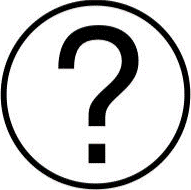 | 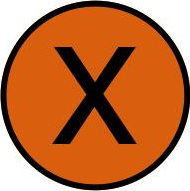 | 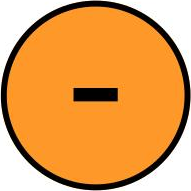 | 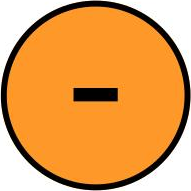 | Critical |
|  | Assume at least, one known important domain was not controlled (adjusted) e.g. MoI, injury severity (and lack of correlation for any other injuries) etc | Selected population (motorcycle accidents only); and potential confounders not adjusted in analyses  Whole of eligible “immobilisation for possible C spine injury “ populations not studied | Intervention defined; and determined retrospectively | No information is reported on whether there is deviation from the intended intervention. | Participants with missing data excluded; no details across interventions and or undertaken appropriate analysis | Assume methods of outcome assessment will be comparable across intervention groups; and  and outcome measure minimally influenced by knowledge of the intervention received by study participants (no indication of blinding); | Outcome measures generally well defined and no indication of selection of results from multiple analyses | At least one domain at critical risk |
| Thompson et al. [Unpublished] | 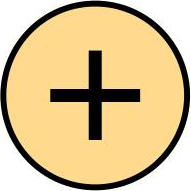 | 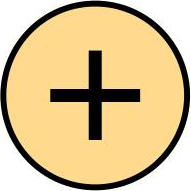 | 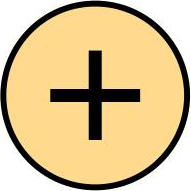 | 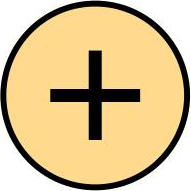 | 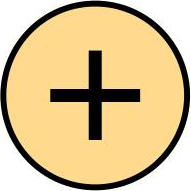 | 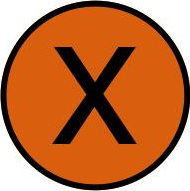 | 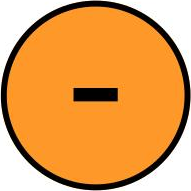 | Serious |
|  | Important domains measured and adjusted for in reporting | Consecutive enrolment of alert stable blunt trauma patients with risk factors for C spine injury in control and intervention periods | Intervention defined and determined prospectively | Deviations from the protocol reported | Patients analysed by study allocation, no missing for primary outcome | No blinding of outcome assessors but methods comparable across groups  Also no information if outcome ascertainment different between groups and for those not having CT; screening tools are not perfect to predict some outcomes | Outcome measures generally well defined and no indication of selection of results from multiple analyses | At least one domain at serious risk |
| Underbrink et al. 2018 [24] | 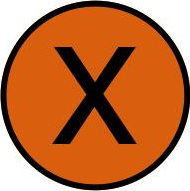 | 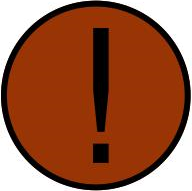 | 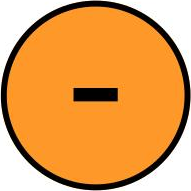 | 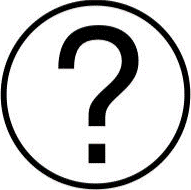 | 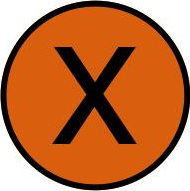 | 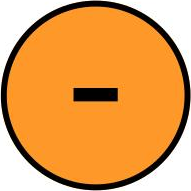 | 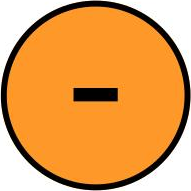 | Critical |
|  | At least, one known important domain was not appropriately measured, or not controlled (adjusted) e.g. MoI | Selected population spinal injury; >60 only; single centre, remote location (different management) in Canada; and not adjusted in analyses, only adjusted by injury severity.  Whole of eligible “immobilisation for possible C spine injury “ populations not studied | Intervention defined; and determined retrospectively (authors note, unclear why some patients received specific immobilisation techniques) | No information is reported on whether there is deviation from the intended intervention (could be possible authors note, unclear why some patients received specific immobilisation techniques) | Participants with missing data excluded; no details across interventions and or undertaken appropriate analysis | Assume methods of outcome assessment will be comparable across intervention groups; and  and outcome measure minimally influenced by knowledge of the intervention received by study participants (no indication of blinding); | Outcome measures generally well defined and no indication of selection of results from multiple analyses | At least one domain at critical risk |

Abbreviations: CT, computed tomography; ED, emergency department; MOI, mechanism of injury


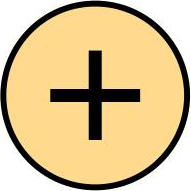
 indicates low risk of bias;
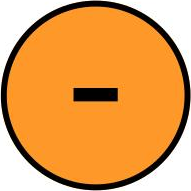
 indicates moderate risk of bias;
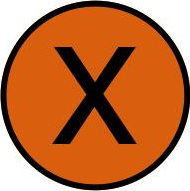
 indicates serious risk of bias;
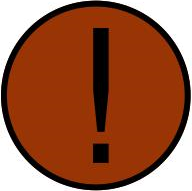
 indicates critical risk of bias and
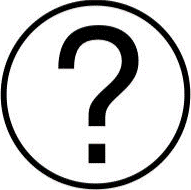
 indicates no information
