# Supplementary material for: Cervical spine immobilisation following blunt trauma in pre-hospital and emergency care: A systematic review
Source: PLoS One. 2024 Apr 25;19(4):e0302127. doi: 10.1371/journal.pone.0302127 (PMC11045128; doi:10.1371/journal.pone.0302127)
Supplement: Attachment — Submitted filename: Pandor et al Response to reviewers.docx [file pone.0302127.s004.docx]

**List of revisions and corrections addressing each of the concerns raised.**

| **Comments** | **Authors’ response** |
| --- | --- |
|  | |
| **Journal requirements** |  |
| 1. When submitting your revision, we need you to address these additional requirements.  Please ensure that your manuscript meets PLOS ONE's style requirements, including those for file naming… | **We can confirm that the manuscript meets PLOS ONE’s style requirements.** |
| 2. Thank you for stating the following in the Acknowledgments Section of your manuscript:  The authors would like to thank all additional members of the core project group for NIHR131430 for input and commentary throughout the work. We are also indebted to Joanne Hinde for assistance with logistics and administration.  We note that you have provided funding information that is not currently declared in your Funding Statement. However, funding information should not appear in the Acknowledgments section or other areas of your manuscript. We will only publish funding information present in the Funding Statement section of the online submission form.   Please remove any funding-related text from the manuscript and let us know how you would like to update your Funding Statement. Currently, your Funding Statement reads as follows:   This study was funded by the United Kingdom National Institute for Health and Care Research Health Technology Assessment Programme (project number 131430).  The views expressed in this paper are those of the authors and not necessarily those of the NIHR or the Department of Health and Social Care. Any errors are the responsibility of the authors. The funders had no role in the study design, in the collection, analysis and interpretation of data; in the writing of the manuscript; and in the decision to submit the manuscript for publication.  Please include your amended statements within your cover letter; we will change the online submission form on your behalf. | **We have updated the Acknowledgements Section and Funding Statement accordingly and added it at the bottom of the cover letter.** |
| 3. Thank you for stating the following in the Competing Interests section:  AP, GF, AS, ME, JS, DSK, MW, GP, PJ, AH, AK, RF and FL all declare funding from NIHR Health Technology Assessment Grant NIHR131430 – Spinal Injury Study. There are no other competing interests.     We note that one or more of the authors are employed by a commercial company: NIHR Health Technology.  a. Please provide an amended Funding Statement declaring this commercial affiliation, as well as a statement regarding the Role of Funders in your study. If the funding organization did not play a role in the study design, data collection and analysis, decision to publish, or preparation of the manuscript and only provided financial support in the form of authors' salaries and/or research materials, please review your statements relating to the author contributions, and ensure you have specifically and accurately indicated the role(s) that these authors had in your study. You can update author roles in the Author Contributions section of the online submission form.  Please also include the following statement within your amended Funding Statement.  “The funder provided support in the form of salaries for authors, but did not have any additional role in the study design, data collection and analysis, decision to publish, or preparation of the manuscript. The specific roles of these authors are articulated in the ‘author contributions’ section.” If your commercial affiliation did play a role in your study, please state and explain this role within your updated Funding Statement.  b. Please also provide an updated Competing Interests Statement declaring this commercial affiliation along with any other relevant declarations relating to employment, consultancy, patents, products in development, or marketed products, etc.  Within your Competing Interests Statement, please confirm that this commercial affiliation does not alter your adherence to all PLOS ONE policies on sharing data and materials by including the following statement: "This does not alter our adherence to  PLOS ONE policies on sharing data and materials.” (as detailed online in our guide for authors <http://journals.plos.org/plosone/s/competing-interests>) . If this adherence statement is not accurate and  there are restrictions on sharing of data and/or materials, please state these. Please note that we cannot proceed with consideration of your article until this information has been declared.  Please include both an updated Funding Statement and Competing Interests Statement in your cover letter. We will change the online submission form on your behalf. | The National Institute for Health and Care Research (NIHR) is a research funding body in the UK and is funded by the UK Department of Health and Social Care to improve the health and wealth of the nation through research. The Health Technology Assessment (HTA) Programme is the largest of the NIHR programmes.  **We have updated the Funding and Competing Interests Statements accordingly and added it at the bottom of the cover letter.** |
| 4. We note that you have referenced Thompson L, C B, Shaw G, McMeekin P, Hawkins C, McClelland G. which has currently not yet been accepted for publication. Please remove this from your References and amend this to state in the body of your manuscript: Thompson L, C B, Shaw G, McMeekin P, Hawkins C, McClelland G. [Unpublished]”) as detailed online in our guide for authors <http://journals.plos.org/plosone/s/submission-guidelines#loc-reference-style> | **We have updated the reference style as suggested**. |
| 5. Please include captions for your Supporting Information files at the end of your manuscript, and update any in-text citations to match accordingly. Please see our Supporting Information guidelines for more information: <http://journals.plos.org/plosone/s/supporting-information>. | **We have updated the Supporting Information style as suggested**. |
|  | |
| **Reviewers' comments** |  |
| **Reviewer #1**  Reviewer #1: This study considers an important and current issue. The study is well conceived, delivered and presented. The data is presented in a logical and accessible fashion. The prose is well written and accessible. | **Thank you for your in-depth review and your comments on the work.**  **We agree this topic is important, very relevant and timely.**  **In response to your suggestions and questions, see our responses below.** |
| One area of feedback relates to clarity in relation to the search strategy and the link to the PROSPERO registration.  - Please confirm if you searched the International Clinical Trials Registry Platform (World Health Organisation from 1990) – I don’t see this in the PROSPERO registration or the supplemental files but in the manuscript | **To clarify, we searched the International Clinical Trials Registry Platform as a grey literature source to identify ongoing and unpublished studies. We believe this is covered in our protocol by the statement "…systematic searches of study registries…".** |
| - Please can you adjust figure 1 to demonstrate where the “additional records found through other sources” (what was the source / which part of the search strategy were these generated by e.g. contacting key experts in the field; and undertaking targeted searches of the World Wide Web using the Google search engine | **Thank you, we have amended Figure 1 accordingly.** |
| Please ensure that critical risk of bias is easily distinguishable from serious risk of bias in all figures. | **The risk of bias figures were generated using the *robvis* software, as noted in our methods section. As we are unable to edit the *robvis* outputs, we have updated the figures using an alternate output format provided by the *robvis* software (i.e. colorblind-friendly scheme).** |
|  | |
| **Reviewer #2**  The objective of this systematic review was to evaluate the impact of different cervical spine immobilization strategies (full immobilization, movement minimization, or no immobilization) on neurological and other outcomes for patients with suspected cervical spinal injury in pre-hospital and emergency department settings. The review included comparative studies examining the benefits and harms of immobilization practices during pre-hospital and emergency care for patients with potential cervical spine injuries following blunt trauma. Six observational studies met the inclusion criteria, but methodological quality varied, with most studies having a serious or critical risk of bias. The evidence did not clearly support the benefit of cervical spine immobilization practices in preventing neurological deterioration, spinal injuries, or death compared to no immobilization. However, collar application during immobilization was associated with increased pain, discomfort, and anatomical complications. The authors concluded that despite limited evidence and weak study designs, pre-hospital cervical spine immobilization may have uncertain value due to a lack of demonstrable benefit and potential complications. They emphasized the need for high-quality comparative studies to address this important question. Indeed, this topic is interesting; however, there are some comments and concerns that should be addressed. | **Thank you for your in-depth review, and your positive comments on the conduct, standard and writing of our work.**  **In response to your suggestions and questions, please see our responses below.**  **We agree this topic is important, very relevant and timely.** |
| Introduction  -While the introduction is detailed, it could benefit from more conciseness. Some sentences are lengthy and complex, making it challenging for the reader to follow the main points.  -Consider breaking down complex sentences into more digestible segments for improved clarity. | **We have shortened sentences as advised for clarity.** |
| -Ensure consistent citation style throughout the introduction. Some citations are provided in square brackets, while others are in regular parentheses. Consistency in citation style is essential for academic writing. | **We can confirm that the manuscript meets PLOS ONE’s style requirements** |
| -The introduction occasionally uses informal language, such as "devastating consequence" and "a devastating impact." While these phrases convey the severity of the issue, consider using more neutral and formal language to maintain an academic tone. | **We have replaced these terms with “life changing” and “major”.** |
| -The transition from discussing the global incidence of spinal cord injuries to the specific focus on cervical spine injuries could be smoother. Consider providing a clearer bridge between these topics for a more seamless flow. | **We have replaced this with “**Though rare, the annual incidence rate varies globally between regions and countries, ranging from 3.6 to 195.4 cases per million population worldwide [2]. Evidence reviews indicate that almost half of all traumatic spinal cord injuries involve the cervical spine, and usually result from cervical spine dislocation [1].“ |
| -Some details, such as the specific equipment used in pre-hospital spinal immobilization, may be more detailed than necessary for the introduction. Consider focusing on essential information to maintain reader engagement and relevance. -The introduction effectively leads to the study's aim, but the sentence introducing the aim is complex. Consider simplifying the sentence structure to clearly convey the research question and objective. | **We have removed some equipment detail whilst retaining the description of triple immobilisation recommended in current guidance.**  **We have simplified this sentence**  “Hence, with this systematic review we aimed to determine if selection of different cervical spine immobilisation practices - during the pre-hospital and emergency department care of patients with possible cervical spine injury - impacts neurological and other outcomes.” |
| -The discussion on the evolving understanding of spinal immobilization is informative, but there is some redundancy in emphasizing the potential harm without introducing new information. Streamline the content to avoid repetition and maintain reader engagement. | **Thank you, we thought it important to detail potential harms to contextualise both current variation in practice and selection of systematic review outcomes.** |
| -Review the text for minor grammatical issues, such as subject-verb agreement and punctuation, to enhance the overall grammatical accuracy. | **This has been done.** |
| Methods The methods section is well-structured and adheres to the standard guidelines for systematic reviews. However, a few suggestions for improvement can be considered: | **Thank you, please see our responses below.** |
| - While the eligibility criteria are generally well-defined, there could be additional clarification on why certain exclusions were made, especially regarding the exclusion of studies involving healthy human volunteers, cadaver or manikin models. Providing a brief rationale for these exclusions would enhance the transparency of the selection process. | **We have now added the following text to in eligibility criteria.** “The systematic review sought to elicit comparative patient and healthcare outcomes associated with different prehospital and emergency department cervical spine immobilisation strategies. Therefore we excluded studies utilising healthy human volunteers (non-trauma), cadaver or manikin models where units of movement or force are often reported but not their effect on clinical outcomes.” |
| - The definition of full cervical spinal immobilization is clear. However, the description of cervical spine management less than triple immobilization is somewhat intricate. Consider simplifying the language for better comprehension, ensuring that readers can easily understand the criteria for studies falling into this category | **We have attempted to simplify this language whilst preserving accuracy** |
| Discussion - The summary of results is clear and concise. However, it might be beneficial to present some key quantitative data, such as the number of studies supporting or refuting the effectiveness of cervical spine immobilization. Providing a brief overview of the characteristics of the included studies (e.g., study design, sample size) could enhance the reader's understanding. | **Thank you, we have added the following text to “interpretation of findings”**  ”None of six observational studies (two prospective and four retrospective - 8207 participants in total) reported any improved outcome associated with a greater degree of cervical spine immobilisation. Five studies reported no difference in comparative rates of cervical spinal cord injury [23, 24, 26, 27] whilst the sixth – at high risk of confounding - reported a significantly higher rate when comparing movement minimisation to no immobilisation [25]. However in the context of uncontrolled heterogeneous studies, we do acknowledge that absence of evidence is not evidence of no benefit.” |
| - While the discussion mentions concerns about pain, discomfort, and anatomical complications associated with collar application during immobilization, consider providing more context on the potential clinical significance of these complications. Discussing the clinical implications will help readers better understand the practical implications of the findings. | **We have added the following text to interpretation of findings**  “There was limited reporting of comparative complications; however, each of the two prospectively recruiting studies found that greater cervical spine immobilisation was associated with higher levels of pain and discomfort. Thompson et al., [Unpublished] also found that as well as distressing immobilised patients this limits adherence, and can also consume scarce ED staff resource - through need for repeated reassurance and/ or prescription and administration of analgesia.” |
| - The comparison to existing literature is informative, but consider discussing any novel or divergent findings in more detail. Highlighting areas where the current review differs from previous ones will contribute to a more nuanced understanding of the research landscape. | **Our review is consistent with the findings of previous reviews cited all of which mention the absence of high quality evidence to support cervical spine immobilisation in blunt trauma – with significant potential harms, We have added to the existing text in “Comparison to the existing literature”** |
| - The strengths and limitations are appropriately discussed. To further enhance transparency, consider providing more details on the rationale behind the selection of the risk of bias tool (ROBINS-I) and why it was deemed appropriate for this review. | **Thank you, we have added the following text to “Strengths and limitations”**  “We also used the ROBINS-I tool, which addresses RoB against an absolute scale rather than using the ideal observational study as a standard [20]. As such, this is the only tool recommended by the Cochrane Collaboration for assessing RoB in non-randomised interventions studies and has been widely adopted for use in published and planned systematic reviews [39].” |
| - The implications for policy and practice are well-discussed. However, consider providing more details on the potential impact of the review findings on clinical guidelines and decision-making in emergency care. Additionally, while the ongoing NIHR Spinal Immobilisation Study is mentioned, consider briefly discussing how its results might influence future practice | **Thank you, we have amended the existing text to read** “Our review provides no clear robust evidence of any protective benefit in routinely immobilising the cervical spine in patients with suspected cervical spine injuries. Clinicians should be aware of this lack of evidence to support the routine and liberal use of cervical spine immobilisation; their clinical decision-making tools should acknowledge this and potential for adverse consequences. However prior to amending existing guidance further research is essential to provide clear evidence-based criteria in applying cervical spine immobilisation. To this end we currently await the results of the ongoing National Institute of Health and Care Research (NIHR) Spinal Immobilisation Study (SIS)….” |
| - The conclusions are clear and aligned with the study findings. To strengthen the conclusion, consider reiterating the key takeaway messages for clinicians and policymakers. Emphasize the need for evidence-based criteria in applying cervical spine immobilization. | **Thank you, we have amended the text of the concluding paragraph accordingly** |
|  | |
